# Managing chemobrain: Translational insights and evidence-based psychological interventions

**DOI:** 10.1017/S1092852926101096

**Published:** 2026-07-29

**Authors:** Tamás Szekeres, Márta Virág, Magdolna Dank, Péter Kovács

**Affiliations:** 1 Rehabilitation Department, National Institute of Oncology, Hungary; 2 Department of Neurointervention and Neurosurgery, Semmelweis University, Hungary; 3 National Institute of Oncology, Hungary; 4 Department of Personality Psychology, Pázmány Péter Catholic University Faculty of Humanities and Social Sciences, Hungary; 5 National Tumor Biology Laboratory, National Institute of Oncology, Hungary

**Keywords:** Chemobrain, neuropsychology, psychological interventions, rehabilitation, psycho-oncology

## Abstract

With the improving survival rates of malignant tumors, the focus of attention in cancer research is shifting toward a better understanding of long-term treatment-related adverse effects that impact quality of life, with particular focus on cancer-related cognitive impairment. This phenomenon, commonly referred to as “chemobrain” in the literature, manifests as deficits in attention, memory, processing speed, and executive functions. Although the frequency of objectively measured cognitive deficits detected through neuropsychological assessments are moderate, subjective complaints often result in significant deterioration of quality of life. The present review aims to provide a multidisciplinary overview of chemobrain, emphasizing its epidemiological characteristics, neurobiological and psychosocial factors, and options for evidence-based psychological interventions. Alongside neurotoxic, inflammatory, hormonal, and neuroendocrine mechanisms associated with chemotherapy, the review highlights distress, cognitive compensation, and discrepancies between subjective complaints and objectively measurable cognitive deficits. Diagnostic challenges and evidence-based psychotherapeutic approaches—especially cognitive behavioral therapy (CBT), mindfulness-based interventions, and rehabilitation—are also discussed. This analysis highlights the complex nature of chemobrain and the need for further research to develop targeted, individualized therapeutic protocols.

## Introduction

The incidence of malignant tumors is increasing globally, while advances in therapeutic approaches—particularly targeted and immuno-oncological treatments—have significantly improved survival outcomes.^1^^,^^2^ According to the most recent GLOBOCAN 2022 estimates, the global burden of cancer continues to increase, with approximately 20 million new cancer cases and 9.7 million cancer-related deaths reported worldwide, highlighting the growing importance of survivorship-related outcomes, including cancer-related cognitive impairment (CRCI).^3^ Consequently, greater attention can now be directed toward long-term quality-of-life consequences, including neurocognitive impairments associated with cancer treatments, the prevalence of which has become a prominent focus of both research and clinical practice in recent years.^4^^,^^5^

Cognitive complaints may emerge shortly after the disclosure of a cancer diagnosis^6^^,^^7^; however, they occur more frequently during and following treatments—particularly chemotherapy.^6^^,^^8^ Although the term “chemobrain” is widely used in both clinical practice and the scientific literature, the broader term CRCI is increasingly preferred. CRCI encompasses cognitive changes associated not only with chemotherapy, but also with cancer itself, endocrine therapy, radiotherapy, surgery, immunotherapy, targeted therapies, inflammation, fatigue, sleep disturbances, psychological distress, comorbidities, and other treatment-related factors. Consequently, CRCI reflects the multifactorial nature of cognitive dysfunctions observed in cancer patients and survivors.^9^^,^^10^ CRCI most commonly affects memory, attention, processing speed, and executive functioning.^11^^,^^12^

Although the prevalence of deficits detectable through neuropsychological assessments are moderate, the frequency of subjective cognitive complaints and their impact on quality of life are considerable.^13^^,^^14^ The discrepancy between subjective and objective measures may be attributable, among other factors, to psychological and socioeconomic influences, as well as to the phenomenon of cognitive compensation.^15^^,^^16^

Given that chemobrain can substantially affect patients’ psychological well-being, work capacity, and social functioning, a more precise understanding and effective management requires a multidisciplinary approach. The aim of this review is to provide an overview of cancer treatment-related cognitive impairments, considering their epidemiological characteristics, underlying pathophysiological and psychological mechanisms, as well as the most relevant evidence-based psychological intervention strategies.

## Literature search strategy

This article was conducted as a narrative review aimed at providing a multidisciplinary overview of CRCI and evidence-based psychological interventions. The literature search was performed using PubMed, Scopus, and Web of Science databases. Key search terms included “chemobrain,” “cancer-related cognitive impairment,” “cognitive dysfunction,” “chemotherapy,” “psychological interventions,” “cognitive rehabilitation,” “mindfulness,” “cognitive behavioral therapy,” “neuroimaging,” and “biomarkers.” Priority was given to systematic reviews, meta-analyses, randomized controlled trials, clinical guidelines, and landmark studies published in English.

The review focused primarily on adult cancer populations receiving systemic anticancer treatment. Studies investigating epidemiology, biological mechanisms, neuropsychological assessment, rehabilitation, and psychological interventions were considered. Due to the predominance of breast cancer research within the CRCI literature, a substantial proportion of the cited studies involve breast cancer survivors. This reflects the current evidence base rather than an intentional restriction of the review scope. Nevertheless, findings from hematological, prostate, testicular, and other cancer populations were included whenever relevant.

## Epidemiological overview

The prevalence of cancer treatment-related cognitive impairments varies widely depending on the type of study, the composition of the patient population, and the methods used to assess cognitive functions. The frequency of subjective cognitive complaints reported in different studies ranges from 20% to 70%, whereas the prevalence of objectively verified cognitive deficits measured by neuropsychological tests is generally lower, typically ranging from 15% to 35%.^5^^,^^13^^,^^17^

The phenomenon has been most frequently investigated in patients with breast cancer, hematological malignancies, and prostate cancer, particularly following adjuvant chemotherapy. A longitudinal study reported that 16% of breast cancer survivors who underwent chemotherapy exhibited moderate to severe cognitive deficits, compared with 5% in the control group.^18^ Another study, conducted more than 20 years after CMF (cyclophosphamide, methotrexate, fluorouracil) treatment, demonstrated a persistent decline in verbal memory, executive functions, and processing.^19^

Interestingly, some cognitive impairments can be detected even before the initiation of treatment, thus likely resulting from psychological distress related to the diagnosis, sleep disturbances, or systemic inflammatory processes. Pretreatment cognitive deficits are often overlooked in cross-sectional studies, which may bias conclusions regarding the effects of chemotherapy.^20^

Among demographic and clinical risk factors such as lower educational attainment, older age, comorbidities (eg, diabetes, cardiovascular diseases), and lower baseline cognitive reserve clearly increase the likelihood of developing cognitive impairments later on.^16^ The type and intensity of treatment may also play a role: certain cytotoxic agents, such as doxorubicin, taxanes, or bendamustine, are associated with greater cognitive dysfunction.^21^

Sex differences are less clear, though more detailed data are available for the female population due to the predominance of breast cancer research. In men, similar cognitive changes have primarily been documented among patients with prostate or testicular tumors, often in association with hormonal treatments.^17^ Several studies examining different types of hormone therapies have partially indicated that tamoxifen use may be associated with cognitive decline, particularly in verbal memory and information processing. However, findings are not entirely consistent, as some studies reported no effect,^22^ while others did (tamoxifen vs. exemestane or tamoxifen vs. letrozole studies).^23^ Not all hormonal treatments show a clear cognitive impact: for example, one study found no significant decline among older breast cancer patients receiving aromatase inhibitor therapy.^24^ Thus, hormonal therapy may potentially contribute to the development of cognitive dysfunction, though its extent and underlying mechanisms remain incompletely understood based on current evidence.

Longitudinal evidence further suggests that cognitive trajectories vary substantially across patients, with some individuals experiencing transient cognitive changes, whereas others report persistent impairments months after treatment completion.^25^

Overall, chemobrain represents a clinically significant phenomenon. Although cognitive symptoms may improve over time in most cases, a substantial proportion of patients experience persistent deficits, particularly during long-term maintenance therapies.^26^ More precise estimates of prevalence and severity require standardized assessment tools, longitudinal studies, and baseline evaluations conducted prior to treatment initiation.

## Biological factors and pathophysiological mechanisms underlying cancer-related cognitive impairment

The development of CRCI is influenced by multiple interacting biological, treatment-related, psychological, and psychosocial factors. Current evidence suggests that CRCI represents a multifactorial condition involving neurotoxicity, neuroinflammatory processes, oxidative stress, genetic susceptibility, hormonal alterations, as well as changes in brain structure and function, which may interact differently across individuals and contribute to the heterogeneity of cognitive outcomes observed among cancer survivors.^9^

### Neurotoxic effects

Several chemotherapeutic agents, particularly platinum compounds, taxanes, anthracyclines, as well as components of the CMF regimen (cyclophosphamide, methotrexate, fluorouracil), are capable of crossing the blood–brain barrier, exerting direct neurotoxic effects on the nervous system. The resulting damage primarily affects the hippocampus, prefrontal cortex, and white matter integrity, which are key regions underlying cognitive functions.^19^

### Neuroinflammation and cytokine release

Chemotherapy induces inflammatory responses, leading to the release of proinflammatory cytokines such as IL-1β, IL-6, and TNF-α. These cytokines can enter the central nervous system and influence neurogenesis, synaptic plasticity, and neurotransmission, thereby contributing to the development of cognitive impairments.^11^^,^^27^^,^^28^

### Glial dysfunction and neuronal damage

Activation of microglial and astrocytic cells leads to the release of neurotoxic mediators, which can cause neuronal damage and synaptic abnormalities.^4^

### Alterations in blood–brain barrier permeability

Certain chemotherapeutic agents are capable of altering the blood–brain barrier permeability, thereby enhancing the penetration of inflammatory and toxic substances into the central nervous system and thus exacerbating neural damage.^29^

### Oxidative stress and mitochondrial dysfunction

Chemotherapy increases oxidative stress in neurons, which can lead to cell death and mitochondrial dysfunction, further impairing cognitive functions.^30^^,^^31^

### Hypothalamic–pituitary–adrenal axis dysfunction

The psychological burden of a cancer diagnosis and its treatments also affects hypothalamic–pituitary–adrenal (HPA) axis functioning, altering cortisol levels and potentially leading to cognitive dysregulation.^32^

### Hormonal changes and endocrine therapy

Chemotherapy-induced early menopause, as well as adjuvant hormonal treatments (eg, tamoxifen, aromatase inhibitors), have been associated with memory decline and declining processing speed.^33^^,^^34^

## Psychological and psychosocial factors

### Anxiety, depression, and fatigue

Psychological distress in cancer patients (eg, fear related to the diagnosis, uncertainty about the future, concerns about treatment side effects) often leads to anxiety and depressive symptoms, which can independently affect cognitive performance—primarily in the domains of attention, concentration, and working memory.^13^^,^^35^

### Subjective versus objective differences

Numerous studies indicate that patient-reported cognitive problems often do not align with objective test results, a discrepancy that may be partly explained by psychological state and distortions in self-perception.^11^^,^^14^

Recent research has further demonstrated that subjective cognitive complaints and objectively measured cognitive performance often show only modest associations in cancer survivors. Subjective complaints appear to be influenced not only by cognitive functioning itself but also by fatigue, anxiety, depression, sleep disturbances, and other psychosocial factors. Consequently, contemporary recommendations emphasize the complementary use of both patient-reported outcomes and objective neuropsychological assessment when evaluating CRCI.^10^

### Cognitive compensation and reserve

According to the cognitive reserve (CR) theory, individuals vary in their ability to compensate for damaging effects. Patients with higher educational attainment, intellect, or socioeconomic status are more likely to compensate for cognitive deficits (eg, through increased neural activation); however, this may also heighten subjective fatigue and the perception of cognitive complaints.^15^^,^^36^

## Assessment of cognitive functions

Accurate identification and documentation of cancer treatment-related cognitive impairments is a complex task, as subjectively perceived complaints often do not align with results from objective neuropsychological assessments.^11^^,^^14^ Consequently, reliable evaluation of cognitive status represents a significant challenge for both research and clinical practice.

### Self-report measures

Assessment of subjective cognitive complaints is often conducted using simple, self-administered questionnaires that can be completed quickly. These tools are sensitive to patient-perceived difficulties—particularly in attention, memory, or executive function—but do not always correlate with objective performance measures.^13^^,^^35^ Subjective complaints can be influenced by a variety of factors, such as anxiety, fatigue, depression, or poor sleep quality.^37^ Nevertheless, these complaints carry significant clinical relevance, as they often better predict declines in quality of life than neuropsychological test results.^38^

### Neuropsychological assessment procedures

A variety of standardized tools are available for the assessment of objective cognitive functions, targeting different cognitive domains. Based on the recommendations of the International Cognition and Cancer Task Force (ICCTF), the following tests are included in the suggested core battery^39^:

- Hopkins Verbal Learning Test–Revised (HVLT-R): verbal learning and memory
- Trail Making Test A and B: processing speed, attention, and executive functions
- Multilingual Aphasia Examination–Controlled Oral Word Association Test (COWAT): verbal fluency

These tools exhibit high reliability and validity and are suitable for detecting mild-to-moderate cognitive impairments associated with cancer treatments. However, it is important to note that practice effects can bias longitudinal results, particularly during over extended follow-up periods.

In recent years, digital cognitive assessment tools and computerized testing platforms have gained increasing attention in psycho-oncology research. These approaches enable standardized administration, automated scoring, repeated longitudinal assessment, and remote monitoring of cognitive functioning. Digital platforms may facilitate the detection of subtle cognitive changes while reducing the logistical burden associated with traditional neuropsychological assessment methods.^40^ Recent evidence further supports the utility of computerized cognitive testing in oncology settings. For example, Gaynor et al. (2024) demonstrated that a computerized neurocognitive battery was sensitive to subtle cancer-related cognitive deficits across multiple domains, including attention, executive functioning, working memory, processing speed, and verbal abilities. Importantly, the battery could also be administered remotely, supporting longitudinal monitoring and improving accessibility for cancer survivors.^41^

Table 1 summarizes the most commonly used neuropsychological tests for assessing chemotherapy-related cognitive impairments, highlighting the targeted cognitive domains, the oncology populations studied, the main findings, and the sensitivity of the assessment tools employed.^9^^,^^22^^,^^25^^,^^39^^,^^41^^-^^48^

**Table 1.** Commonly Used Assessment Tools for the Evaluation of Cancer-Related Cognitive Impairment (CRCI), Including Cognitive Domains Assessed, Clinical Applications, and Principal Strengths Table 1. long description.

Assessment tool	Category	Cognitive domain(s) assessed	Typical use in CRCI assessment	Key strength
HVLT-R	Core neuropsychological test	Verbal learning, immediate and delayed memory	Recommended as part of the ICCTF core battery	Sensitive to verbal memory deficits frequently observed in CRCI
TMT-A/B	Core neuropsychological test	Processing speed, attention, cognitive flexibility, executive functioning	Recommended as part of the ICCTF core battery	Brief administration and sensitivity to executive dysfunction
COWAT	Core neuropsychological test	Verbal fluency, lexical retrieval	Recommended as part of the ICCTF core battery	Sensitive to deficits in verbal fluency and executive language functions
SCWT	Additional neuropsychological test	Selective attention, inhibitory control, executive functioning	Frequently used in CRCI research protocols	Assesses cognitive inhibition and interference control
CD, SS	Additional neuropsychological test	Processing speed, attention, visuomotor integration	Common measure of cognitive processing speed	Sensitive to subtle slowing in cognitive performance
RBANS	Additional neuropsychological test	Attention, memory, language, visuospatial abilities	Broad screening and multidomain assessment	Evaluates multiple cognitive domains within a single battery
BNT	Additional neuropsychological test	Language and naming abilities	Assessment of language-related cognitive functioning	Sensitive to word-finding difficulties
GPT	Additional neuropsychological test	Psychomotor speed, fine motor dexterity	Assessment of motor functioning and treatment-related psychomotor slowing	Useful when neuropathy may influence cognitive performance
CANTAB	Computerized cognitive assessment	Working memory, learning, attention, executive functioning	Research and longitudinal monitoring	Standardized computerized administration and multidomain assessment
CBB	Computerized cognitive assessment	Attention, learning, memory, reaction time	Repeated assessment of cognitive functioning	Minimal practice effects and suitability for longitudinal follow-up
ANT	Computerized cognitive assessment	Alerting, orienting, executive attention	Research assessment of attentional networks	Detailed evaluation of attentional processes
CCTB	Computerized cognitive assessment	Multiple cognitive domains	Remote and repeated assessment of CRCI	Automated scoring, remote administration, and longitudinal monitoring
FACT-Cog	Patient-reported outcome measure	Perceived cognitive functioning and impact on daily life	Assessment of subjective cognitive complaints	Captures patient-reported cognitive difficulties and quality-of-life impact

*Note:* Assessment tools are categorized according to their primary methodological approach and include objective neuropsychological measures, computerized cognitive assessments, and patient-reported outcome measures. Cognitive domains and clinical applications are summarized based on published validation studies and CRCI research recommendations.

Abbreviations: HVLT-R, Hopkins Verbal Learning Test–Revised; TMT-A/B, Trail Making Test; COWAT, Controlled Oral Word Association Test; SCWT, Stroop Color–Word Test; CD, Digit Symbol-Coding; SS, Symbol Search; RBANS, Repeatable Battery for the Assessment of Neuropsychological Status; BNT, Boston Naming Test; GPT, Grooved Pegboard Test; CANTAB, Cambridge Neuropsychological Test Automated Battery; CBB, Cogstate Brief Battery; ANT, Attention Network Test; CCTB, Computerized Cognitive Test Batteries; FACT-Cog, Functional Assessment of Cancer Therapy—Cognitive Function.

### Biomarkers and neuroimaging techniques

Functional neuroimaging techniques, such as fMRI and EEG, offer the opportunity to objectively observe cognitive compensation mechanisms and changes in brain activity. In patients who have undergone chemotherapy, increased prefrontal activation has been observed during verbal memory tasks, which may indicate effortful processing and compensation by other brain regions.^49^ However, these methods are not currently part of routine clinical diagnostics and are primarily limited to research settings. Furthermore, a standardized biomarker to support the diagnosis of CRCI is not yet available.

Beyond their diagnostic value, biomarkers and neuroimaging findings may also have important translational implications for personalized management of CRCI. Emerging evidence suggests that inflammatory markers (eg, IL-6, TNF-α), neuroendocrine alterations, and neuroimaging patterns reflecting changes in functional connectivity or compensatory neural activation could help identify patients at increased risk of persistent cognitive dysfunction and support risk stratification efforts. Recent studies indicate that inflammatory biomarkers, particularly IL-6, may have predictive value for long-term cognitive outcomes, while neuroimaging markers may contribute to the identification of biologically distinct cognitive phenotypes.^40^^,^^50^

Future precision-medicine approaches are expected to integrate biological, neuropsychological, neuroimaging, and psychosocial data to develop individualized treatment pathways. Combining biomarker assessment with cognitive screening, patient-reported outcomes, and predictive models may allow clinicians to identify vulnerable patients before treatment initiation and tailor supportive interventions accordingly. Although these approaches remain largely investigational, they represent a promising direction for translating mechanistic insights into clinically meaningful management strategies for chemobrain.^40^^,^^50^^,^^51^

Recent research has also focused on emerging biological markers that may improve the understanding and prediction of CRCI. In particular, inflammatory pathways remain a major area of interest, with accumulating evidence suggesting that persistent systemic inflammation may contribute to long-term cognitive dysfunction.^52^ Furthermore, advances in neuroimaging research have identified structural and functional brain alterations that may serve as potential biomarkers of cognitive vulnerability and recovery.^40^ Blood-based biomarkers, including markers of neuroaxonal injury, have also attracted increasing attention as potential tools for monitoring treatment-related neurotoxicity and cognitive outcomes.^40^

Another emerging area concerns the gut–brain axis. Alterations in the gut microbiome may influence neuroinflammatory processes and neural signaling pathways that are relevant to cognitive functioning. Although evidence in oncology populations remains preliminary, microbiome-related mechanisms have been proposed as potential contributors to CRCI and warrant further investigation.^40^

### Diagnostic challenges

A primary challenge is the integration of discrepancies between patient-reported cognitive complaints and objectively measured deficits into clinical decision making. To address this issue, several authors advocate for the concurrent use of both subjective and objective assessments, complemented by preassessment evaluation of mood and sleep quality.^14^^,^^16^

## Psychotherapeutic interventions for chemobrain: Evidence and guideline perspectives

The management of chemobrain requires a multifaceted approach, in which psychotherapeutic interventions play a key role, particularly in alleviating common psychological symptoms in cancer patients, such as anxiety, depression, and cognitive complaints.^4^ Contemporary reviews indicate that several nonpharmacological interventions show promise in the management of CRCI; however, methodological limitations and variability across studies continue to limit definitive conclusions regarding their effectiveness.^53^

Numerous clinical studies have demonstrated that cognitive behavioral therapy (CBT) is effective in improving psychological well-being and supporting cognitive functioning in cancer patients.^20^ Among CBT-based approaches, Memory and Attention Adaptation Training (MAAT) has received particular attention. MAAT is a structured intervention specifically developed for cancer survivors experiencing chemotherapy-related cognitive difficulties. The program combines psychoeducation, self-monitoring of cognitive lapses, stress-management techniques, cognitive restructuring, and compensatory memory and attention strategies. In a randomized wait-list controlled trial, participants receiving MAAT reported significant improvements in quality of life and perceived cognitive functioning compared with controls, though evidence regarding long-term durability remains limited.^54^

Although the current evidence generally supports the use of psychological interventions for CRCI, the strength of evidence varies across intervention types. CBT demonstrated beneficial effects on subjective cognitive complaints, psychological distress, fatigue, and quality of life; however, improvements in objectively measured cognitive performance have been less consistent across studies. Similarly, mindfulness-based interventions have shown small-to-moderate effects on perceived cognitive functioning and emotional well-being, though long-term follow-up data remain limited.^55^^,^^56^

Cognitive rehabilitation programs currently have the strongest direct focus on cognitive outcomes. Several randomized controlled trials have reported improvements in attention, memory, executive functioning, and compensatory strategy use following structured cognitive rehabilitation interventions.^29^^,^^56^ Nevertheless, substantial heterogeneity exists regarding intervention content, duration, outcome measures, and follow-up periods, making direct comparisons across studies difficult.

In contrast, evidence for mindfulness-based interventions is generally characterized by small-to-moderate effects on subjective cognitive complaints and psychological distress, whereas effects on objective cognitive performance are less consistent. Similarly, cognitive rehabilitation programs have shown promising results, but substantial heterogeneity in intervention content, outcome measures, and follow-up periods limits direct comparison across studies.^56^

Importantly, relatively few studies have examined the durability of treatment effects beyond 6–12 months, and evidence regarding long-term maintenance of cognitive gains remains limited. Consequently, while current findings are promising, additional large-scale randomized controlled trials with standardized outcome measures and extended follow-up periods are required before definitive recommendations regarding specific intervention protocols can be established.^56^

Sleep disturbances are highly prevalent among cancer survivors and have been associated with subjective cognitive complaints and reduced neurocognitive functioning.^57^ Emerging evidence suggests that cognitive behavioral therapy for insomnia (CBT-I) may represent an important adjunctive intervention, as improvements in sleep quality are often accompanied by reductions in fatigue, anxiety, and other factors that contribute to cognitive difficulties.^58^ Recent randomized controlled trial evidence further supports this approach. Garland et al. (2024) demonstrated that virtually delivered CBT-I significantly improved perceived cognitive functioning among cancer survivors, suggesting that targeting sleep disturbances may represent an effective strategy for addressing subjective CRCI symptoms.^59^

Mindfulness-based stress reduction (MBSR) programs have also been shown to improve perceived cognitive functioning, emotional regulation, and psychological well-being, though effects on objective neuropsychological performance appear less consistent across studies.^55^ Furthermore, structured cognitive rehabilitation programs, which combine psychotherapeutic elements with cognitive training, have proven to be effective in mitigating cognitive deficits and improving daily functioning.^4^^,^^29^

Recent evidence from a network meta-analysis suggests that neuropsychological interventions may improve selected CRCI outcomes; however, effects vary across intervention modalities and cognitive domains. Cognitive rehabilitation and cognitive training interventions appear to show greater benefits for objective cognitive outcomes, whereas psychological interventions may exert stronger effects on subjective cognitive complaints and psychosocial functioning.^60^ Similarly, a recent network meta-analysis focusing on breast cancer survivors concluded that nonpharmacological interventions may improve CRCI-related outcomes, though the magnitude and consistency of effects differed substantially across intervention types and outcome measures.^61^

Current international clinical guidelines, including those from the European Society for Medical Oncology (ESMO) and the National Institute for Health and Care Excellence (NICE), do not yet provide explicit psychotherapeutic recommendations specifically targeting chemobrain. These guidelines broadly recommend monitoring cognitive function in cancer patients and ensuring psychosocial support, with particular emphasis on the management of anxiety and depression.^62^^,^^63^ While psychotherapeutic interventions such as CBT and mindfulness-based techniques are suggested for the mitigation of psychological distress, a standardized, chemobrain-specific protocol has not yet been established.

Cochrane database meta-analyses also indicate that, though cognitive rehabilitation and psychotherapeutic programs show promise in improving cognitive functions, the current evidence is insufficient to formulate standardized treatment guidelines.^56^ Consequently, further large-scale, randomized controlled trials are needed to develop effective, individualized therapeutic protocols.

A recent network meta-analysis focusing on breast cancer survivors similarly concluded that nonpharmacological interventions may improve CRCI-related outcomes, though the magnitude and consistency of effects differed substantially across intervention types and outcome measures.^61^

Overall, current evidence suggests that psychotherapeutic and behavioral interventions may contribute to the management of CRCI; however, their effects appear to vary according to intervention type, outcome domain, and patient population. Psychological interventions such as CBT and mindfulness-based approaches demonstrate the most consistent benefits for perceived cognitive difficulties, psychological distress, coping, and quality of life, whereas cognitive rehabilitation and cognitive training interventions may provide greater benefits for selected objective cognitive domains, including attention, memory, and executive functioning. Nevertheless, the available evidence remains heterogeneous, and further high-quality randomized controlled trials are required to establish optimal intervention protocols and determine long-term effectiveness.

## Guideline recommendations for the management and rehabilitation of chemobrain

The management of chemobrain, or chemotherapy-induced cognitive impairment, is not currently encompassed by distinct, explicit protocols within international clinical guidelines. However, guidelines issued by the ESMO and NICE provide comprehensive recommendations for the cognitive and psychosocial support of cancer patients, which may also be applicable to the management of chemobrain.^62^^,^^63^

Guidelines already emphasize the importance of regular monitoring of cognitive function in cancer patients, particularly following the completion of chemotherapy, to enable timely identification and management of cognitive complaints.^62^ The implementation of a multidisciplinary approach is recommended, involving clinicians, psychologists, neuropsychologists, and rehabilitation specialists to address complex cognitive and psychosocial issues.^63^

Within the framework of psychosocial support, clinical guidelines advocate for the management of anxiety and depression, which commonly co-occur with cognitive symptoms and can further exacerbate them. CBT and mindfulness-based stress reduction interventions have demonstrated efficacy in this context, particularly in mitigating psychological distress.^62^^,^^63^

Cognitive rehabilitation programs, encompassing structured cognitive training and memory enhancement exercises, are likewise recommended for the improvement of cognitive function. However, guidelines emphasize that, in the absence of additional large-scale randomized controlled trials, the current evidence remains insufficient to support the development of standardized protocols.^56^

Moreover, the promotion of lifestyle interventions—including regular physical activity, adherence to a healthy diet, sufficient sleep, and effective stress management—represents a fundamental strategy for the maintenance and enhancement of cognitive function.^63^ Growing evidence suggests that structured exercise and physical rehabilitation programs may contribute to the prevention and management of CRCI. Recent systematic reviews and meta-analyses have reported beneficial effects of aerobic exercise, resistance training, and combined exercise interventions on self-reported cognitive functioning, executive functions, and cognitive fatigue among cancer survivors.^64^^,^^65^ Although the magnitude of effects varies across studies and cancer populations, exercise is increasingly recognized as a promising nonpharmacological component of comprehensive oncology rehabilitation. These recommendations are consistent with recent practical guidance for oncologists, which emphasizes routine assessment of cognitive symptoms, identification and management of contributing factors such as anxiety, depression, fatigue, and sleep disturbances, promotion of physical activity and mindfulness-based approaches, implementation of compensatory cognitive strategies, and referral to cognitive rehabilitation services when appropriate.^10^

In conclusion, both international and national guidelines currently endorse a comprehensive, multidisciplinary, and individualized rehabilitation strategy for addressing cognitive complaints in cancer patients.^62^^,^^63^^,^^66^ This approach may also prove beneficial for mitigating the symptoms of chemobrain until specific treatment guidelines are formally established.

## Summary

With the significant improvement in survival rates for malignant cancers, there has been an increasing research and clinical focus on long-term, quality-of-life–impacting effects, particularly CRCI, often referred to in the international literature as chemobrain. These cognitive alterations manifest as both quantitative and qualitative declines in attention, memory, processing speed, and executive functions. Although the prevalence of objectively detected deficits via neuropsychological assessments is moderate, patient-reported subjective complaints frequently correlate with substantial reductions in quality of life.

Despite the heterogeneity in the epidemiological data, the prevalence of subjective cognitive complaints ranges from 20% to 70%, whereas objectively measured neuropsychological deficits occur in approximately 15% to 35% of patients. The pathogenesis of cancer treatment-related cognitive impairment involves complex, interacting biological mechanisms—including direct neurotoxicity, neuroinflammation, hormonal dysregulation, oxidative stress, and HPA axis dysfunction—as well as psychological and psychosocial determinants such as anxiety, depression, and cognitive reserve. A major challenge in clinical practice is the inconsistency between subjective and objective cognitive measures, highlighting the need for integrated, multimodal assessment protocols.

Based on the available evidence, psychological interventions—primarily CBT, mindfulness-based programs, and structured cognitive rehabilitation—have been shown to positively influence both cognitive performance and psychological well-being. However, leading international clinical guidelines currently do not provide explicit, cancer treatment-specific protocols for managing cognitive impairment, offering only general recommendations for longitudinal monitoring of cognitive functions, provision of psychosocial support, and incorporation of preventive lifestyle interventions. The development of targeted, individualized therapeutic algorithms necessitates multidisciplinary research efforts, standardized assessment tools, and findings from large-scale randomized controlled trials.

## Long descriptions

### Table 1. Long description

The table consists of five columns and thirteen rows of data.

Row 1: H V L T dash R. Category: Core neuropsychological test. Domains: Verbal learning, immediate and delayed memory. Use: Recommended as part of the I C C T F core battery. Strength: Sensitive to verbal memory deficits frequently observed in C R C I.

Row 2: T M T dash A forward slash B. Category: Core neuropsychological test. Domains: Processing speed, attention, cognitive flexibility, executive functioning. Use: Recommended as part of the I C C T F core battery. Strength: Brief administration and sensitivity to executive dysfunction.

Row 3: C O W A T. Category: Core neuropsychological test. Domains: Verbal fluency, lexical retrieval. Use: Recommended as part of the I C C T F core battery. Strength: Sensitive to deficits in verbal fluency and executive language functions.

Row 4: S C W T. Category: Additional neuropsychological test. Domains: Selective attention, inhibitory control, executive functioning. Use: Frequently used in C R C I research protocols. Strength: Assesses cognitive inhibition and interference control.

Row 5: C D, S S. Category: Additional neuropsychological test. Domains: Processing speed, attention, visuomotor integration. Use: Common measure of cognitive processing speed. Strength: Sensitive to subtle slowing in cognitive performance.

Row 6: R B A N S. Category: Additional neuropsychological test. Domains: Attention, memory, language, visuospatial abilities. Use: Broad screening and multidomain assessment. Strength: Evaluates multiple cognitive domains within a single battery.

Row 7: B N T. Category: Additional neuropsychological test. Domains: Language and naming abilities. Use: Assessment of language-related cognitive functioning. Strength: Sensitive to word-finding difficulties.

Row 8: G P T. Category: Additional neuropsychological test. Domains: Psychomotor speed, fine motor dexterity. Use: Assessment of motor functioning and treatment-related psychomotor slowing. Strength: Useful when neuropathy may influence cognitive performance.

Row 9: C A N T A B. Category: Computerized cognitive assessment. Domains: Working memory, learning, attention, executive functioning. Use: Research and longitudinal monitoring. Strength: Standardized computerized administration and multidomain assessment.

Row 10: C B B. Category: Computerized cognitive assessment. Domains: Attention, learning, memory, reaction time. Use: Repeated assessment of cognitive functioning. Strength: Minimal practice effects and suitability for longitudinal follow-up.

Row 11: A N T. Category: Computerized cognitive assessment. Domains: Alerting, orienting, executive attention. Use: Research assessment of attentional networks. Strength: Detailed evaluation of attentional processes.

Row 12: C C T B. Category: Computerized cognitive assessment. Domains: Multiple cognitive domains. Use: Remote and repeated assessment of C R C I. Strength: Automated scoring, remote administration, and longitudinal monitoring.

Row 13: F A C T dash Cog. Category: Patient-reported outcome measure. Domains: Perceived cognitive functioning and impact on daily life. Use: Assessment of subjective cognitive complaints. Strength: Captures patient-reported cognitive difficulties and quality-of-life impact.

Navigate back to Table 1..
